# Discovering Clinical Information Models Online to Promote Interoperability of Electronic Health Records: A Feasibility Study of OpenEHR

**DOI:** 10.2196/13504

**Published:** 2019-05-28

**Authors:** Lin Yang, Xiaoshuo Huang, Jiao Li

**Affiliations:** 1 Institute of Medical Information / Medical Library Chinese Academy of Medical Sciences & Peking Union Medical College Beijing China

**Keywords:** openEHR, clinical information model, health information interoperability, information retrieval, probabilistic graphical model

## Abstract

**Background:**

Clinical information models (CIMs) enabling semantic interoperability are crucial for electronic health record (EHR) data use and reuse. Dual model methodology, which distinguishes the CIMs from the technical domain, could help enable the interoperability of EHRs at the knowledge level. How to help clinicians and domain experts discover CIMs from an open repository online to represent EHR data in a standard manner becomes important.

**Objective:**

This study aimed to develop a retrieval method to identify CIMs online to represent EHR data.

**Methods:**

We proposed a graphical retrieval method and validated its feasibility using an online CIM repository: openEHR Clinical Knowledge Manager (CKM). First, we represented CIMs (archetypes) using an extended Bayesian network. Then, an inference process was run in the network to discover relevant archetypes. In the evaluation, we defined three retrieval tasks (medication, laboratory test, and diagnosis) and compared our method with three typical retrieval methods (BM25F, simple Bayesian network, and CKM), using mean average precision (MAP), average precision (AP), and precision at 10 (P@10) as evaluation metrics.

**Results:**

We downloaded all available archetypes from the CKM. Then, the graphical model was applied to represent the archetypes as a four-level clinical resources network. The network consisted of 5513 nodes, including 3982 data element nodes, 504 concept nodes, 504 duplicated concept nodes, and 523 archetype nodes, as well as 9867 edges. The results showed that our method achieved the best MAP (MAP=0.32), and the AP was almost equal across different retrieval tasks (AP=0.35, 0.31, and 0.30, respectively). In the diagnosis retrieval task, our method could successfully identify the models covering “diagnostic reports,” “problem list,” “patients background,” “clinical decision,” etc, as well as models that other retrieval methods could not find, such as “problems and diagnoses.”

**Conclusions:**

The graphical retrieval method we propose is an effective approach to meet the uncertainty of finding CIMs. Our method can help clinicians and domain experts identify CIMs to represent EHR data in a standard manner, enabling EHR data to be exchangeable and interoperable.

## Introduction

Electronic health record (EHR) data can be used and reused for many purposes, including managing an individual patient’s care, medical and health services research, and management of health care facilities. More recently, EHR data has been defined as a part of real-world data [1] and is increasingly seen as a viable source of data for regulatory decisions [2]. However, bias can occur in different steps of the data chain, which might lead to incomparable or invalid analysis results [3].

Semantic interoperability is essential for accurate and advanced health-related computing, shared EHRs, and coordination of clinical care across clinical systems [4,5]. According to ISO/TS 18308 (a standard published by the International Organization for Standardization defining the set of requirements for EHR architecture), it is the ability for data shared by systems to be understood at the level of fully defined domain concepts [6]. To achieve this, a two-level clinical modeling methodology is proposed to separate clinical knowledge from information models [7]. It distinguishes two models: the reference model (RM), which contains the basic and stable properties of health record information, and the clinical information model (CIM), which formally defines clinical concepts (or domain content models) in a standardized and reusable manner, such as blood pressure [8,9]. In this scenario, CIMs in agreement at an organizational, regional, national, or international level will provide a firm basis for establishing semantic interoperability [9].

This two-level modeling approach is used in the ISO/CEN EN13606 (a standard designed to achieve semantic interoperability in EHR communication) [10] and openEHR (described subsequently) [11], as well as Health Level Seven (HL7) version 3 Clinical Document Architecture (HL7's primary standard for representing structured clinical documentation on patients) and Care Provision messages (information structures used to communicate information between providers of care) [12]. For openEHR and ISO/CEN EN13606, CIMs are defined in the form of archetypes, whereas those of HL7 are in the form of HL7 templates. According to the systematic review done by Moreno-Conde et al [13], archetypes are the preferred type of technical artifacts, and openEHR is most frequently mentioned. Therefore, CIMs in our study specifically refer to openEHR archetypes.

OpenEHR is an open-source EHR standard ensuring universal interoperability among all forms of electronic data [14-21]. It is well known for its two-level design paradigm, consisting of an RM, archetypes, and templates. *Archetypes* are computable clinical content specifications that formalize the patterns and requirements for the representation of health information content [9]. To achieve common, coherent, and clinician-approved archetypes, the openEHR community provides a Web-based controlled authoring environment for a wide range of domain experts, especially clinicians, to participate in the creation of archetypes. All contributions are open access and freely available under a Creative Commons license. Archetypes are general purpose, reusable, and composable; therefore, searching for reusable archetypes from archetype repositories is essential throughout the development process [22,23]. Documents with complete archetype design specifications are the input; lists of existing reusable archetypes, either complete or needing modifications, and new archetypes to be developed from scratch are the output [23]. The crucial problem is how to find the relevant ones from open repositories to help identify reusable archetypes.

The openEHR community provides the Clinical Knowledge Manager (CKM) [24] to be a library of openEHR archetypes. It supports their retrieval based on clinical concepts in different sections of archetypes. When the end user enters a term, the CKM will return the archetype that contains the word in metadata, definition, or ontology section. It could help find reusable archetypes [25]. However, domain experts are mainly concerned about whether the concept name and core data items are covered [17,26,27], and they may be not familiar with openEHR archetypes, especially clinicians. For better results, end users usually need to do a large amount of preparatory work, which may include classifying and rearranging data [27], abstracting clinical concepts from data schemas [17], and identifying archetype-friendly concepts from clinical statements [26]. It is an iterative and time-consuming process.

We aimed to develop a retrieval method to identify archetypes online to represent EHR data and optimize existing retrieval results of the CKM. Archetypes usually have their own hierarchical structures, and semantic relationships occur between different archetypes; therefore, we considered that the graphical representation of this potential knowledge might support the retrieval of CIMs. Previous studies show that graphs could efficiently represent clinical knowledge [28-30], and the Bayesian network, as a probabilistic graphical model, is an effective methodology to meet the uncertainty of information needs. Rotmensch et al [30] used a naive Bayes classifier and a Bayesian network to automatically construct a health knowledge graph from electronic medical records. However, in retrieval tasks, differences between Bayesian network-based information retrieval methods mainly lie in the structure of the network, and this structure depends on dependencies between the variables involved in the problem. The basic Bayesian network consists of two different sets of variables, a set of indexing terms and a set of documents in the collection, and the relationships between them [31]. Related research has been conducted to extend a simple Bayesian network for better results. Some methods focus on the structure of the term subnetwork using a polytree [32,33] or two term layers [34,35] to represent term relationships. Some focus on the structure of the document subnetwork using two document layers [36] to represent document relationships. Compared with the previous studies, we focused on the probabilistic graphical representation of openEHR archetype sets, which depends on relationships between the variables involved in finding relevant archetypes, and how the inference process is carried out, aiming for better retrieval performance.

## Methods

### Information Need Analysis

To find relevant archetypes from the open repository, we first had to understand which kinds of terms end users tended to enter. As archetype modeling methodology [23] shows, domain experts identify core clinical concepts and related data elements involved in a particular scenario and organize them into mind maps or design tables. These archetype design specifications are the main source of search keywords. We considered that the input of end users was mainly the names of clinical concepts or related data elements.

Ideally, the user enters the clinical concept and the system feeds back the archetype defining the concept, or the user enters data elements related to a concept and the system feeds back the archetype that covers all the data elements. However, it is difficult to distinguish clinical concepts and data elements from the end user’s input, unless it forces users to input separately. More importantly, data elements defined by end users may be the concept in an archetype repository, or the defined concept is the data element of an archetype. If we match concepts and data elements separately, users may miss some important relevant archetypes.

Based on these considerations, we tried to translate the problem into identifying potentially relevant clinical concepts from the input. We proposed to reorganize the archetype collection with the dependencies between clinical concepts, data elements, and archetypes and used a probabilistic approach to meet the uncertainty of user information needs.

### Graphical Retrieval Method Based on an Extended Bayesian Network

#### Archetype Feature Identification and Extraction

**Figure 1:**
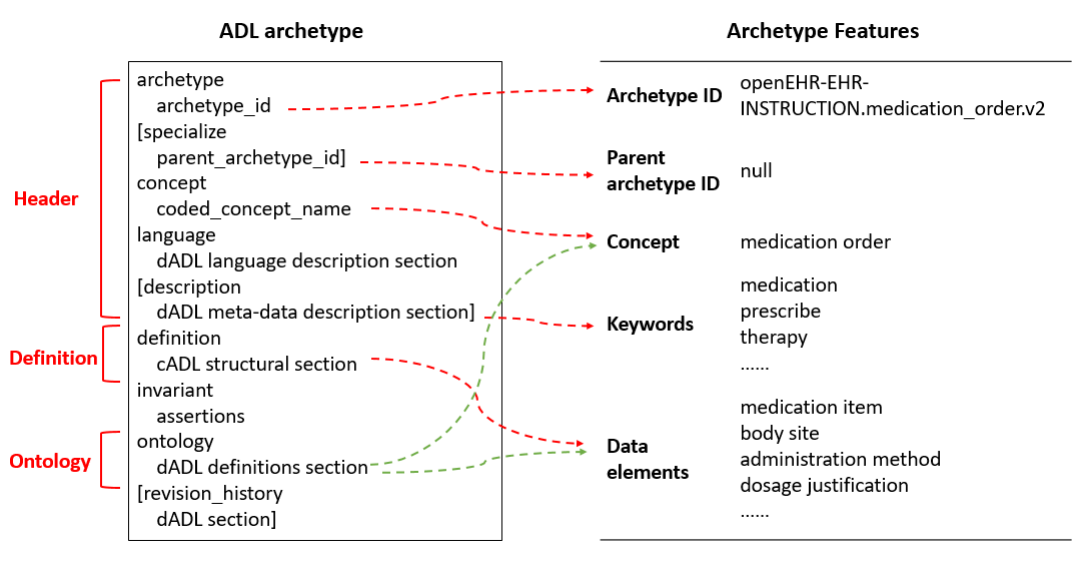
An example of archetype feature identification and extraction.

Based on information need analysis, we attempted to use clinical concepts and data elements to represent each archetype. An archetype is expressed in Archetype Definition Language (ADL) and mainly consists of three sections (Figure 1). The header contains a unique identifier for the archetype and includes some descriptive information, such as concept name and keywords; the definition contains the main formal definition of the archetype, including all possible data elements that could be relevant for the clinical concept; and the ontology contains the code that represents the meaning of nodes. We considered that clinical concepts were the topics of archetypes, whereas keywords and data elements explained the meaning of topics from different perspectives. Thus, we extracted archetype ID, concepts, keywords, and data elements based on ADL files parsing as features (Figure 1).

There are also relationships between archetypes, including specialization and aggregation. An archetype is a specialization of another if it mentions that archetype as its parent and only makes changes to its definition. Aggregation enables any subset of archetypes to be stated as the allowed set for use in a compositional parent archetype. In general, archetypes tend to provide highly reusable models of real-world content with local constraining left to templates, which may result in matching as many archetypes as possible when defining archetype slots. For example, “openEHR-EHR-CLUSTER.device_details.v1” allows the inclusion of 199 archetypes. We thought that such cases might blur the semantic relationship between archetypes. In addition, version control is an integral part of the openEHR architecture. When an archetype updates, the old version could not be found in the archetype library. Therefore, we only added the parent archetype ID as the feature (Figure 1).

Furthermore, there are four main categories of archetypes, including COMPOSITION, SECTION, ENTRY, and CLUSTER, each defined as part of the openEHR RM. A COMPOSITION is a container class, whereas a SECTION is an organizing class, each containing ENTRY objects [16]. The ENTRY class is further specialized into ADMIN_ENTRY, OBSERVATION, EVALUATION, INSTRUCTION, and ACTION subclasses, of which the latter four are kinds of CARE_ENTRY. CLUSTERS are reusable archetypes for use within any ENTRY or other CLUSTER. In addition, the openEHR designs Demographic archetypes for demographic information. Thereby, archetypes could be mainly divided into COMPOSITION, SECTION, ENTRY, CLUSTER, and DEMOGRAPHIC. However, these archetype categories will not obscure the clinical content, and we did not use these as the feature.

#### Clinical Resources Network Modeling

**Figure 2:**
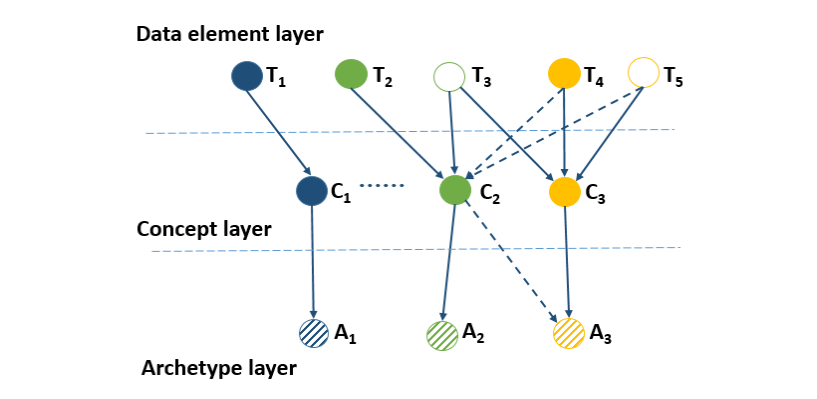
Topology of three-level clinical resources network. A: archetype; C: clinical concept; T: data element.

We attempted to use a three-level Bayesian network to represent the dependencies among data elements, concepts, and archetypes (Figure 2). The first is the data element layer. It contains the set of indexing data elements T={*T*_i_, i=1...M}, *M* being the number of data elements from a given archetype collection. Each data element node is linked to its corresponding concept node in the clinical concept layer. The second is the clinical concept layer. It contains the set of indexing concepts C={*C*_j_, j=1...N}, *N* being the number of concepts. The third layer contains the set of archetypes A={*A*_k_, k=1...K}, *K* being the total number of archetypes in the collection. If *A*_k_ is a specialization of another archetype *A*_p_ which defines *C*_j_, there is a link joining any concept node *C*_j_ and any archetype node *A*_k_.

However, data elements are unevenly distributed across different types of archetypes, especially for container classes. When two archetypes have few data elements and terms used are totally different, such as “openEHR-EHR-COMPOSITION .medication_list.v0” and “openEHR-EHR-SECTION.medication _order_list.v0,” it is difficult to find correlation between them.

**Figure 3:**
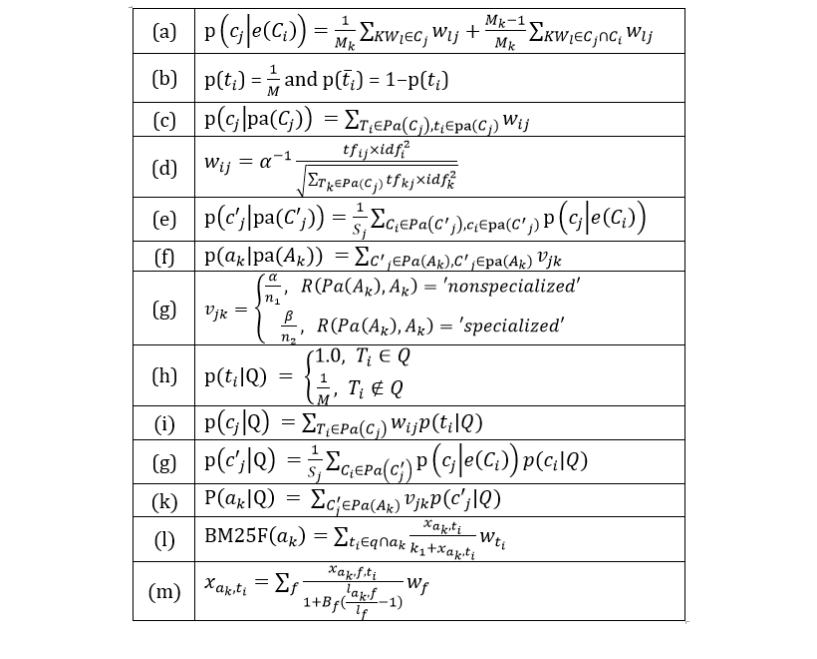
Equations used in our method.

Therefore, we tried to include relationships between concepts in the model to extend the similarity between archetypes. Relationships between concepts were measured by estimating conditional probabilities of relevance of every concept given that another concept was considered relevant [36]. Let *e* (*C*_i_) be an event representing some type of evidence about the relevance of a concept *C*_i_. In openEHR, the evidence could be “keywords,” “purpose,” “use,” or other semantic information. In this case, we considered that *e* (*C*_i_) as the event [*KW*_l_= *kw*_l,_ ∀ *KW*_l_∈ *C*_i_], *KW* being the keywords used to describe the concept. Given a concept *C*_j_, we calculated the probabilities *p* (*c*_j_| *e* (*C*_i_)) ∀ *C*_i_∈ *C* using equation (a) in Figure 3, where the weight was computed by equation (d) in Figure 3 and *M*_k_ was the number of keywords. After decreasing the ordering of *p*(*c*_j_|*e*(*C*_i_)), the top n concepts *R*_n_(*C*_j_) were the ones that were more related to *C*_j_. Then, we included in the network-explicit dependence relationships between *C*_j_ and each concept *C*_i_∈*R*_n_(*C*_j_).

To determine the topology of the Bayesian network, we used a concept subnetwork with two layers instead of the original concept layer. We duplicated each concept node *C*_j_ to obtain another concept node *C*^ʹ^_j_, thus forming a new concept layer, and the arcs connecting the two layers went from *C*_i_∈*R*_n_(*C*_j_) to *C*^ʹ^_j_. Thus, this directed acyclic graph had the set of variables V=T∪C∪C^ʹ^∪A. The new topology avoids connections between nodes in the same layer and facilitates the inference process.

**Figure 4:**
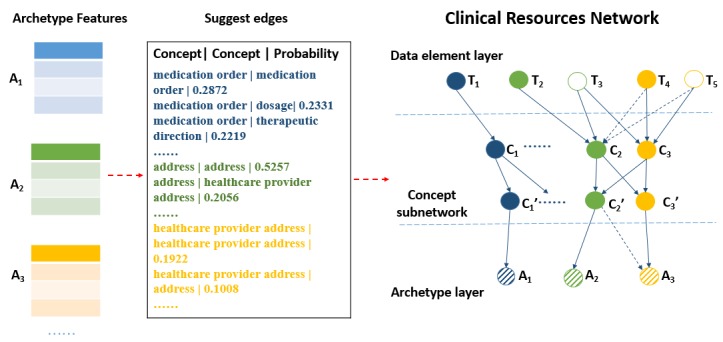
Clinical resources network modeling pipeline. A: archetype; C: clinical concept; Cʹ: duplicated clinical concept; T: data element.

The overall modeling procedure is summarized in Figure 4. First, we extracted archetype ID, clinical concept, and data elements from the ADL files (detailed in section “archetype feature identification and extraction”). Second, we learned the dependencies between concepts (detailed previously). Third, we graphically represented the dependencies between the variables.

#### Parameters Estimation in the Clinical Resources Network

In this section, we will discuss how to estimate the probability distributions of each node in the network.

##### Data Element Nodes

A data element node has no parents; therefore, we had to store the probability of relevance *p* (*t*_i_) and the probability of being nonrelevant. We used the estimator (Figure 3, equation b), where *M* is the number of terms used to index the concept collection.

##### Concept Nodes

For each concept node *C*_j_ in the concept subnetwork, we needed to estimate a set of conditional probability distributions *p* (*c*_j_|*pa*(*C*_j_)). *Pa*(*C*_j_) represents the parent nodes set of concept *C*_j_, containing all the data elements belonging to concept *C*_j_, and *pa* (*C*_j_) is a possible configuration of value associated with the parent set *Pa*(*C*_j_). We used the estimator (Figure 3, equations c and d) proposed by De Campos et al [33], where α is a normalizing constant (assure ∑_Ti__∈__Pa(Cj)_*w*_ij_≤1 ∀ *C*_j_*∈* *C*), *tf*_ij_ is the term frequency of data element *T*_i_ in concept *C*_j_, and *idf*_i_ is the inverse concept frequency of *T*_i_ in the whole concept collection; *idf*_i_ = *1* + *log* (*N* / *n*_i_), N being the total number of concepts, and *n*_i_ being the total number of concepts containing *T*_i_.

For each concept node *C*^ʹ^_j_, we need to estimate a set of conditional probability distributions *p*(*c*^ʹ^_j_|pa(*C*^′^_j_)). We used the estimator (Figure 3, equation e) proposed by Acid et al [36], where *S*_j_ = ∑_Ck__∈__Pa(C′j)_*p*(*c*_j_|*e*(*C*_k_)) and the values *p*(*c*_j_|*e*(*C*_k_)) are obtained when modeling the network.

##### Archetype Nodes

For each archetype node *A*_k_, we needed to estimate a set of conditional probability distributions *p*(*a*_k_| *pa*(*A*_k_)). *Pa*(*A*_k_) represents the parent node sets of archetype *A*_k_, containing all the concepts belonging to archetype *A*_k_, and *pa*(*A*_k_) is a possible configuration of values associated with the parent set *Pa* (*A*_k_). *v*_jk_ is a constant to represent the weight of a concept for an archetype. The estimator is shown in Figure 3, equations (f) and (g), where *R*(*Pa*(*A*_k_), *A*_k_) represents two different relationships between the concept and archetype, *n*_1_ is the number of “nonspecialized” archetypes of one concept, and *n*_2_ is the number of “specialized” archetypes, whereas α and β are coefficients for the weight.

#### Relevant Archetype Discovering: Inference in the Clinical Resources Network

To find relevant archetypes is to estimate the probability of relevance *p* (*a*_k_|Q) for each archetype, *Q* being an end user query.

Given a query *Q*, the set of terms used to formulate the query will be a new piece of evidence. The retrieval process starts by placing the evidence in the data element subnetwork. Then, the inference process is run in the clinical resources network. This allows us to obtain the probability of relevance of each archetype, given that the terms in the query are relevant, *p* (*a*_k_|Q). Finally, the archetypes will be sorted in decreasing order of probability to carry out the evaluation process. The inference process is composed of four stages.

1. Terms in the data element layer are marginally independent; therefore, the probability of relevance *p*(*t*_i_|Q) is calculated by equation (h) in Figure 3.
2. Based on the propagation process, the conditional probability of concept *C*_j_ in the concept subnetwork for the query Q could be calculated by equation (i) in Figure 3.
3. The conditional probability of concept *C*^ʹ^_j_ in the concept subnetwork for the query Q could be computed using equation (g) in Figure 3.
4. The conditional probability of archetype *A*_k_ for the query Q, *p*(*a*_k_|Q) could be carried out using information obtained in the previous step by the equation (k) in Figure 3.

Therefore, the propagation with this topology is to evaluate equations (h), (i), (g), and (k) in Figure 3.

### Experiment Setup

#### Test Queries

We defined test queries with the following considerations: first, clinical concepts to be retrieved should be essential components of the EHR; second, there should be needs to reuse these clinical contents [37], such as medical events prediction [38], clinical research [39], and disease research [40]; third, queries should allow us to test the performance of retrieval methods in related archetypes identification, including specialized archetypes and compositional parent archetypes. Based on these criteria, we selected medication, laboratory test, and diagnosis as retrieval tasks and formulated three queries (Table 1).

#### Data Source

We downloaded all available archetypes from the CKM [24] for a total of 526 on August 30, 2018. All files were in ADL format. We used the ADL parser [41] to extract features. Among these CIMs, three archetypes did not use English as the description language, so the total number changed to 523.

#### Relevance Assessment

To evaluate retrieval results, we first had to identify relevant archetypes in three retrieval tasks as the gold standard. We manually annotated all 523 archetypes, according to their relevance to each query, to formulate three benchmark datasets. Given a query and an archetype, three annotators were asked to judge if the archetype was relevant. The labeling instructions were as follows: a label was relevant when the archetype could cover the potential clinical concept inferred from the given query; a label was nonrelevant otherwise. We took the majority vote to decide the relevance of an archetype. These three benchmark datasets were used as ground truth for the medication, laboratory test, and diagnosis retrieval tasks.

#### Baseline Methods

To validate the performance of our method, three typical retrieval methods were selected as baselines: CKM, BM25F, and simple Bayesian network.

**Table 1:** Test queries.

Query	Retrieval task	Input terms
1	Medication	Medicine name, total daily amount, allowed period, and order start date/time
2	Laboratory test	Report, test name, and test results
3	Diagnosis	Problem/diagnosis, test diagnosis, date/time of onset, and body site

BM25F is an extension of the BM25 ranking function, which is applicable to structured documents consisting of multiple fields. It combines the term frequencies (weighted accordingly to their field importance) and uses the resulting pseudofrequency in the BM25 ranking function. In this study, we supposed that an archetype was decomposed into two fields, concept and data elements, and used the function (Figure 3, equations l and m) proposed by Zaragoza et al [42], where *w*_ti_ is the RSJ relevance weight for term *t*_i_, *x*_ak, f, ti_ is the term frequency of term *t*_i_ in the field type *f* of archetype *a*_k_, *l*_ak, f_ is the length of that field, *l*_f_ is the average field length for that field type, and *B*_f_ is a field-dependent parameter.

For the Bayesian network, the structure is illustrated in Figure 2. The propagation with this topology is to evaluate equations (h), (i), and (k) in Figure 3.

## Results

### Overview of Clinical Resources Network

Table 2 shows the distribution of archetypes across different clinical domains.*Clinical domain classification* refers to the concept schema proposed by Hruby et al [39].

Table 3 shows the distribution of archetypes, concepts, and data elements across different types of archetypes in the collection. In addition, there were 31 specialized archetypes, 11 of whose parent archetypes are no longer in the CKM.

Then, we learned the dependencies between concepts. Table 4 shows the top relevant concepts suggested by four different percentages of values of *p*(*c*_j_|*e*(*C*_i_)) for “dosage” and “examination of a lung,” respectively.

After that, we constructed four clinical resource networks, G_1_, G_2_, G_3_, and G_4_, according to the top 3%, 5%, 8%, and 10% of values, respectively. Each graph consisted of 5513 nodes, which were 3982 data element nodes, 504 concept nodes, 504 duplicated concept nodes, and 523 archetype nodes, with 6366 edges from T to C and 543 edges from Cʹ to A. For edges C to Cʹ, G_1_ had 1590 arcs, G_2_ had 2485 arcs, G_3_ had 2958 arcs, and G_4_ had 3263 arcs.

### Evaluation of the Performance

To compare the performance of different graphs in supporting retrieval, we calculated the average precision (AP) values for the 11 standard recall points of each graph for the test queries and then computed the mean average precision (MAP) values. The results (Table 5) showed that the retrieval method based on G_3_ achieved the best MAP (MAP=0.32), with an AP of 0.35, 0.31, and 0.3 for each query, respectively.

**Table 2:** Distribution of archetypes across different clinical domains.

Clinical domain and subdomains		Archetypes, n
**Patient**		
	Demographic	42
	Health characteristic	32
	Patient	6
**Pretreatment diagnosis**		
	Clinical assessment	73
	Pretreatment diagnosis	26
	Procedure	6
	Intent	1
**Treatment**		
	Treatment	39
	Prescribed	12
	Surgery	9
Detection/Treatment results		184
Organizational/Provider characteristics		26
Outcomes		24
Patient environment factors		6
Other		37
Total		523

**Table 3:** Distribution of archetypes, concepts, and data elements.

Archetype type subtypes		Archetypes, n	Concepts, n	Elements, n	Data elements per concept, mean
Cluster		198	198	1567	7.9
Composition		25	25	45	1.8
**Entry**					
	Action	15	15	252	16.8
	Evaluation	51	51	432	8.5
	Observation	164	163	1511	9.3
	Instruction	8	8	124	15.5
	Admin	4	4	69	17.3
Section		26	26	88	3.4
Demographic		32	29	169	5.8
Total		523	504	3982	7.9

**Table 4:** Top edge suggestions for “dosage” and “examination of lung.”

Clinical concept	Different threshold of *p*(*c*_j_|*e(C*_i_))^a^			
	Top 3%	Top 5%	Top 8%	Top 10%
Dosage	Dosage	Dosage	Dosage	Dosage
	Medication order	Medication order	Medication order	Medication order
		Therapeutic direction	Therapeutic direction	Therapeutic direction
			Medication	Medication
			Medication authorization	Medication authorization
Examination of lung	Examination of a lung	Examination of a lung	Examination of a lung	Examination of a lung
	Auscultation of lung	Auscultation of lung	Auscultation of lung	Auscultation of lung
	Pulmonary function test	Pulmonary function test	Pulmonary function test	Pulmonary function test
	Macroscopic findings-lung cancer	Macroscopic findings-lung cancer	Macroscopic findings-lung cancer	Macroscopic findings-lung cancer
				Examination findings-posterior chamber of eye
				Examination of a breast
				Examination of a burn

^a^c_j_=”dosage” and “examination of lung,” respectively.

Next, we compared the results of our method based on G_3_ with baseline methods. To comprehensively validate the performance, we selected the MAP, AP, and precision at 10 (P@10) as evaluation metrics. Archetypes in the CKM are updated regularly, so it is difficult for us to compare the result on the same collection. We searched relevant archetypes in the CKM for the three queries given on December 12, 2018, and evaluated its performance against the ground truth. The result (Table 6) shows that our method outperforms all the baseline methods, achieving the best AP and P@10 across different test queries, as well as the best MAP. For instance, for query 1, our method, CKM, Bayesian network, and BM25F achieved a P@10 of 0.50, 0.40, 0.20, and 0.20, respectively. Furthermore, we can observe that the MAP of BM25F (MAP=0.177) and Bayesian network (MAP=0.127) was lower than that of CKM (MAP=0.227), which means that there are limitations in using clinical concepts and data elements to represent each archetype. Our approach takes into account the semantic associations between concepts and effectively compensates for this deficiency.

**Figure 5:**
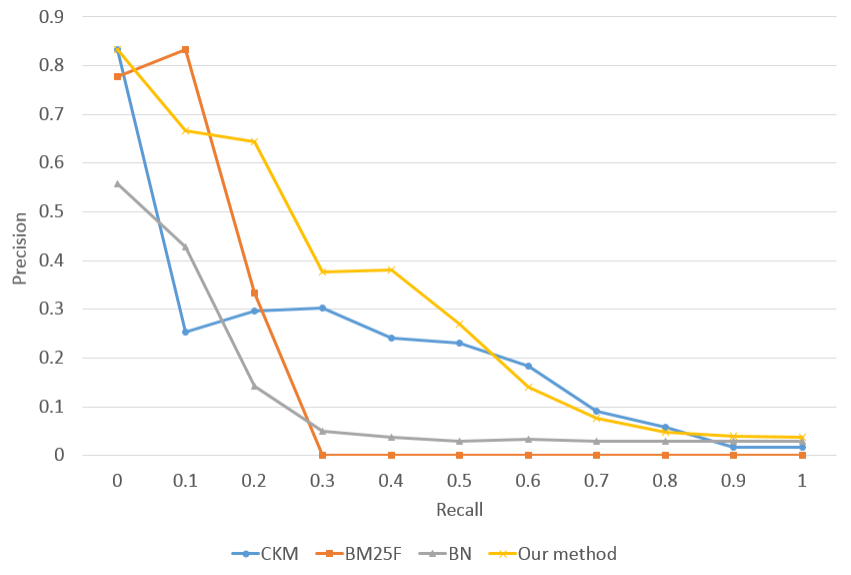
Precision-recall curves of the four retrieval methods. BM25F: an extension of the BM25 ranking function; BN: Bayesian network; CKM: Clinical Knowledge Manager.

The same trend is observed when evaluating precision-recall graphs across all test queries. Figure 5 shows the precision-recall curves evaluated against the ground truth. Here, BM25F falls short in performance. For instance, for a recall of 0.3, our method, CKM, Bayesian network, and BM25F achieved a precision of 0.38, 0.30, 0.05, and 0, respectively. Additionally, the 11-point MAP curve of the Bayesian network is similar to that of our approach, but the performance is much worse than ours. Meanwhile, compared with the curve of the CKM, our curve is smoother and has higher precision when the recall is below 0.6. These results may be explained by the fact that dependencies between concepts could help identify relevant archetypes.

**Table 5:** Average precision performance of graphs with different similarity thresholds.

Graphs with different similarity thresholds^a^	Mean average precision	Average precision		
		Query 1 (medication)	Query 2 (laboratory test)	Query 3 (diagnosis)
G_1_ (top 3%)	0.253	0.36	0.10	0.30
G_2_ (top 5%)	0.277	0.27	0.26	0.30
G_3_ (top 8%)	0.320	0.35	0.31	0.30
G_4_ (top 10%)	0.313	0.33	0.31	0.30

^a^Graphs with percentages of values of *p*(*c*_j_|*e(C*_i_)).

**Table 6:** Retrieval performance comparison.

Method	MAP^a^	Query 1 (medication)		Query 2 (laboratory test)		Query 3 (diagnosis)	
		AP^b^	P@10^c^	AP	P@10	AP	P@10
CKM	0.227	0.26	0.40	0.31	0.30	0.11	0.10
BM25F	0.177	0.08	0.20	0.18	0.30	0.27	0.30
Bayesian network	0.127	0.11	0.20	0.22	0.30	0.05	0.10
Our method	0.320	0.35	0.50	0.31	0.50	0.30	0.30

^a^MAP: mean average precision.

^b^AP: average precision.

^c^P@10: precision at 10.

## Discussion

### Principal Findings

The dual model methodology used by openEHR distinguished the clinical content domain from the technical domain, which enabled reusable CIMs (archetypes) [9]. We were interested in identifying relevant CIMs online to standardize clinical concept representation within EHRs, so we developed a graphical retrieval method based on an extended Bayesian network and validated its feasibility using an online clinical information knowledge source: OpenEHR CKM. We combined a qualitative representation of the retrieval task, by using a graphical representation of relationships among data elements, concepts, and archetypes, with quantitative representation of the uncertainty of information needs, using a probabilistic approach. Compared with three typical retrieval methods (BM25F, Bayesian network, and CKM) in the medication, laboratory test, and diagnosis retrieval tasks, our method achieved the best MAP (MAP=0.32). In the diagnosis retrieval task, CKM and BM25F could not find the relevant archetype “openEHR- EHR-SECTION.problems_and_diagnoses.v1.” Our method could successfully identify the models covering “diagnostic reports,” “problem list,” “patients background,” “clinical decision,” etc, as well as “problems and diagnoses.”

Although end users were mainly concerned about whether an archetype covered the concept name and core data items, we could not obtain satisfied performances without considering any potential knowledge that might be mined from the collection. Here, BM25F and Bayesian network just used clinical concepts and data elements as main features to represent each archetype and performed worse compared with the other models. In the laboratory test retrieval task, the recall of BM25F was 0.158, whereas ours was 1.0 and CKM was 0.895. In the diagnosis retrieval task, the value of precision at 3 of Bayesian network was 0, whereas ours was 1.0 and CKM was 0.333. A possible reason was that we used exact matching instead of fuzzy matching. The most important reason was that they only encoded the dependence relationships between variables and did not take into account the semantic associations between them. Previous studies showed that using the structure of existing knowledge resources and distributional statistics drawn from text corpora could help estimate semantic similarity and relatedness between medical concepts [43]. In the openEHR framework, archetypes should map to clinical terminologies (such as SNOMED CT). However, most archetypes currently in the CKM lacked this kind of mapping, which could have limited the calculation of semantic relatedness. In this study, we learned relationships between concepts by a probabilistic approach and constructed a concept subnetwork with two layers. The results showed that the performance significantly improved, which explained the effectiveness of using prior knowledge to improve retrieval results.

Accordingly, how to find the top *n* concepts relevant with each concept became crucial. We used e(*C*_i_) as an event representing some type of evidence about the relevance of a concept *C*_i_, and keywords were used as evidence in the experiment. With their help, we could find that the concepts “medication list” and “medication order list” were related, even though their concept name and data elements were totally different. There was also other semantic information that could be used as evidence, such as “purpose” and “use.” How to use them to better support retrieval might need to be further clarified. However, this method could also include in the network some lower relevant concepts, as shown in the column “Top 10%” in Table 4. For better results, we used AP and MAP as evaluation metrics to help select relevant concepts; meanwhile, we noticed that many concepts had the same values of conditional probabilities. This was because of the probabilistic approach we applied, which reminded us that we could not simply select the top *n* concepts as the relevant ones. Here, we adopted concepts with top *n* percentages of values of conditional probabilities.

When modeling clinical resources network, we took the relationship of specialization between archetypes into consideration. It helped us find “openEHR-EHR- COMPOSITION.report-result.v1,” a specialized archetype of “openEHR-EHR- COMPOSITION.report.v1,” which BM25F could not find. In addition, we could also find relevant compositional parent archetypes successfully, even though we did not use the relationship of aggregation. For example, in the diagnosis retrieval task, our method could find “openEHR-EHR-SECTION.clinical_decision.v0,” which defined an archetype slot to allow “openEHR-EHR- EVALUATION.problem_diagnosis.v1.” It was because the compositional archetype used the clinical concept of the allowed archetype as its data element. When we linked the data element node to its corresponding concept node, we in fact modeled the relationship of aggregation.

The key idea of our approach lay in identifying potentially relevant clinical concepts from the input. In a two-level model methodology, clinicians were usually the end users. In most scenarios, they were not familiar with openEHR archetypes and did not know what archetype-friendly concepts were. This requires the retrieval method to be as insensitive to the input as possible. For example, take the medication retrieval task. If the user inputs “medication item, order start date/time, dosage, dose unit, comment,” using some frequent words in the archetype library, the CKM performed better than ours. The AP value of CKM was 0.82 (P@10=0.7, recall=1) whereas ours was 0.45 (P@10=0.6, recall=1). However, when the user used uncommon words, such as “medicine name” (Table 1), our method, CKM, Bayesian network, and BM25F achieved an AP of 0.35, 0.26, 0.11, and 0.08, respectively. In addition, as Table 6 shows, our AP was almost equal across different retrieval tasks (0.35, 0.31, and 0.30, respectively), whereas the other retrieval methods were not. From the clinical domain, queries 2 and 3 mainly belonged to the topic of detection/treatment results, whereas query 1 belonged to treatment, which indicated that our performance was relatively stable across different clinical domains. All these showed that our method was more robust than the others.

Additionally, better retrieval results could help users to identify reusable archetypes quickly, promote reuse of archetypes, and improve standardization of CIMs, thereby enhancing interoperability of EHRs. Archetype modeling methodology [15,23] showed that clinicians and domain experts should compare archetype design specifications with retrieved archetypes to decide whether new archetypes need to be developed or whether an existing one could be adapted. Our method could successfully identify relevant archetypes that the CKM could not find, such as “openEHR-EHR- SECTION.problems_and_diagnoses.v1” in the diagnosis retrieval task. If this archetype was the case need, domain experts might create a new one as they thought it did not exist in the CKM. Our method achieved the best recall (recall=1) in different retrieval tasks, which could help reuse archetypes and promote the semantic interoperability of EHRs.

### Limitations

Our study has important limitations. First, it is a feasibility study based on openEHR archetypes. Whether our method can be applied to other CIMs, such as HL7 templates, and to what extent it needs to be localized still need to be clarified and validated. In fact, the key features used in our method are data elements, clinical concepts, CIMs (archetypes), and their relationships. It indicates that our method has potential feasibility if these features are available for other CIMs. Which results are potentially possible will be discussed in future work.

Second, our method presented in this study lacks the calculation of the semantic relevance of synonyms or homonyms, both for queries and network modeling. However, relevant semantic computing methods [43] can be applied to our retrieval method. With their help, we may be able to identify that “medication item” and “medicine item” referred to the same term, and the results would be improved. In the future, we will validate its feasibility and effectiveness.

Third, we did not validate the impact of our method on interoperability. In fact, the basic problem of semantic interoperability in EHRs must be solved from the perspective of the business domains the concepts originally belong to. Our approach only addresses specific technical issues in the CIM modeling process.

Furthermore, there are other limitations. First, the relevant archetypes in the three retrieval tasks that we manually annotated may be controversial, according to different experts. Second, we compared our performances with the CKM on different archetype collections, which may lead to inaccurate results.

### Conclusions

In this paper, we proposed an extended Bayesian network retrieval method for finding relevant CIMs. We graphically represented openEHR archetypes using an extended Bayesian network with two concept layers. The results show that it is an effective approach to meet the uncertainty of retrieval tasks, and the key step in modeling this network is to learn the dependencies between concepts. Our better retrieval results could encourage clinicians and domain experts to reuse existing CIMs to represent EHR data in a standard manner, thereby enhancing the interoperability of EHRs. Furthermore, our study provided how the inference process was carried out. Comparing the results of our method with baseline methods, we had the best performance. To optimize the method, further research should focus on the potential feasibility for other CIMs and the calculation of semantic relevance of synonyms or homonyms.

## Abbreviations

ADL: Archetype Definition Language
AP: average precision
CIM: clinical information model
CKM: Clinical Knowledge Manager
EHR: electronic health record
HL7: Health Level Seven
MAP: mean average precision
P@10: precision at 10
RM: reference model
